# Supplementary material for: Vesicular release probability sets the strength of individual Schaffer collateral synapses
Source: Nat Commun. 2022 Oct 17;13:6126. doi: 10.1038/s41467-022-33565-6 (PMC9576736; doi:10.1038/s41467-022-33565-6)
Supplement: Supplementary file 1 — Supplementary Information [file 41467_2022_33565_MOESM1_ESM.pdf]

Supplementary Information

for

Vesicular release probability sets the strength of individual Schaffer collateral synapses

Céline D. Dürst, J. Simon Wiegert, Christian Schulze, Nordine Helassa, Katalin Török and Thomas G. Oertner

Inventory

Supplementary Figure 1: Temporal stability of iGluSnFR transients.

Supplementary Figure 2: Measured release probabilities are independent of imaging noise.

Supplementary Figure 3: Two sigma threshold to discriminate successes from failures.

Supplementary Figure 4: Multisynaptic boutons show a second release site under high *p*r.

Supplementary Figure 5: Low occupancy of AMPARs.

Supplementary Figure 6: Unchanged synaptic release probability upon block of NMDAR.

Supplementary Figure 7: Expression of iGluSnFR does not affect synaptic transmission.

Supplementary Figure 8: Histogram bin size effects.

Supplementary Figure 9: Extracted parameters are robust to assumed saturation value.

Supplementary Figure 10: Correlation matrix of extracted quantal parameters.

Supplementary Figure 11: Extracting iGluSnFR kinetics from synaptic imaging data.

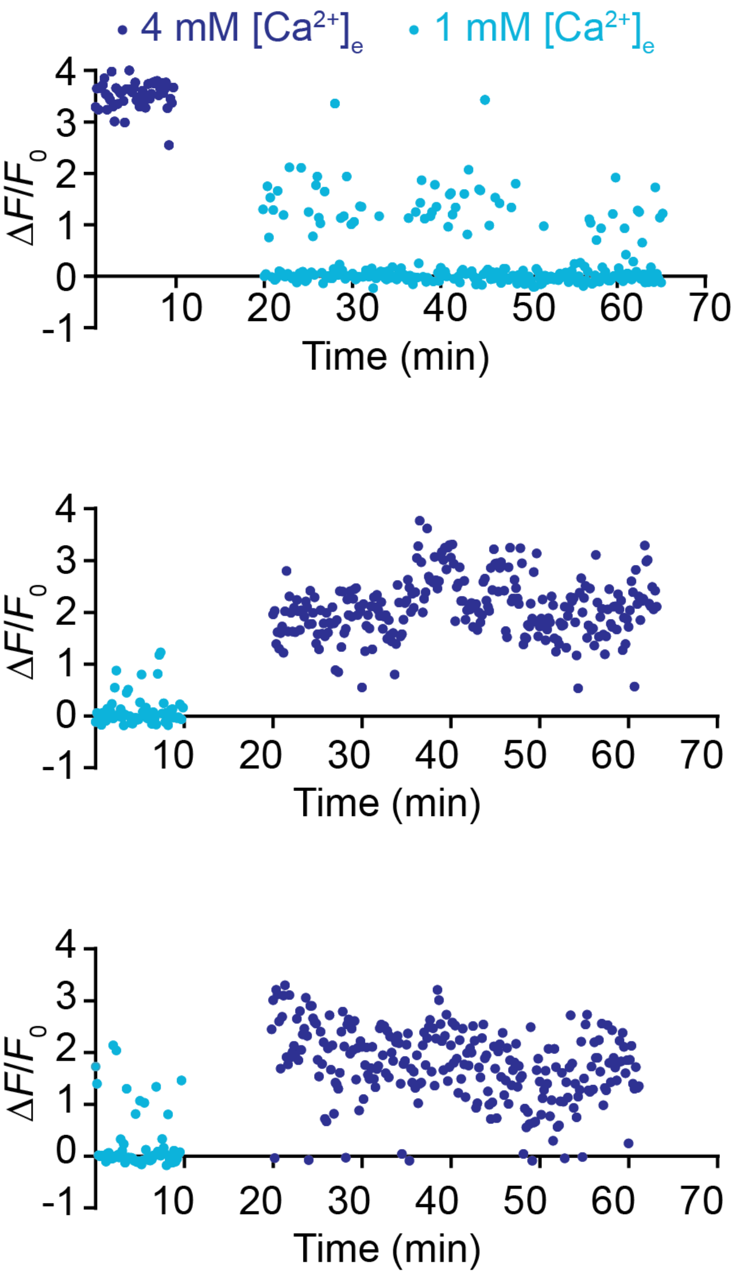

Supplementary Figure 1: Temporal stability of iGluSnFR transients.
Response amplitude in response to single presynaptic APs monitored in ACSF containing 1 and 4 mM $[Ca^{2+}]_e$ at 33°C. During the wash-in period (10 min) from 1 to 4 mM $[Ca^{2+}]_e$ or vice versa, no images were acquired. Three different Schaffer collateral boutons from 3 different organotypic cultures. Source data are provided as a Source Data file.

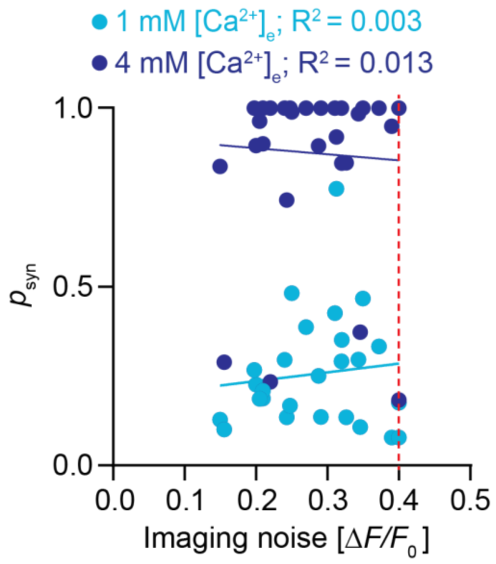

Supplementary Figure 2: Estimated release probabilities were independent of imaging noise. p_{syn} plotted as a function of the imaging noise. The imaging noise corresponds to the full width at half maximum (FWHM) of a Gaussian fit to the distribution of the baseline noise from trial to trial for a given boutons. Experiments with an imaging noise above a FWHM of 0.4 $\Delta F/F_0$ (red dotted line) were discarded. Source data are provided as a Source Data file.

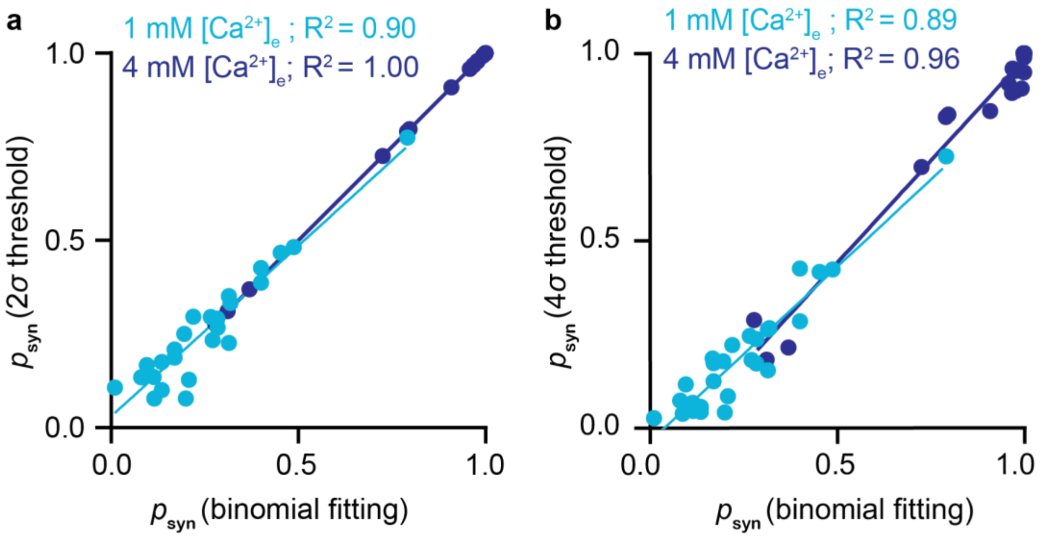

Supplementary Figure 3: Two sigma threshold to discriminate successes from failures delivers p_{syn} values consistent with full quantal analysis.

a) Synaptic release probability (p_{syn}) measured as the number of successes (responses exceeding 2σ of baseline fluorescence fluctuations) divided by the number of trials, plotted as a function of p_{syn} calculated from the quantal parameters extracted with the binomial fitting procedure ($p_{syn} = 1-(1-p_{ves})^N$) (Equation 5). Linear fits show high correlation between the two estimates of p_{syn}. **b)** Same as **a**, but classified with a higher threshold (4σ). The resulting estimates of p_{syn} are generally lower

and not as consistent with our binomial quantal analysis. Source data are provided as a Source Data file.

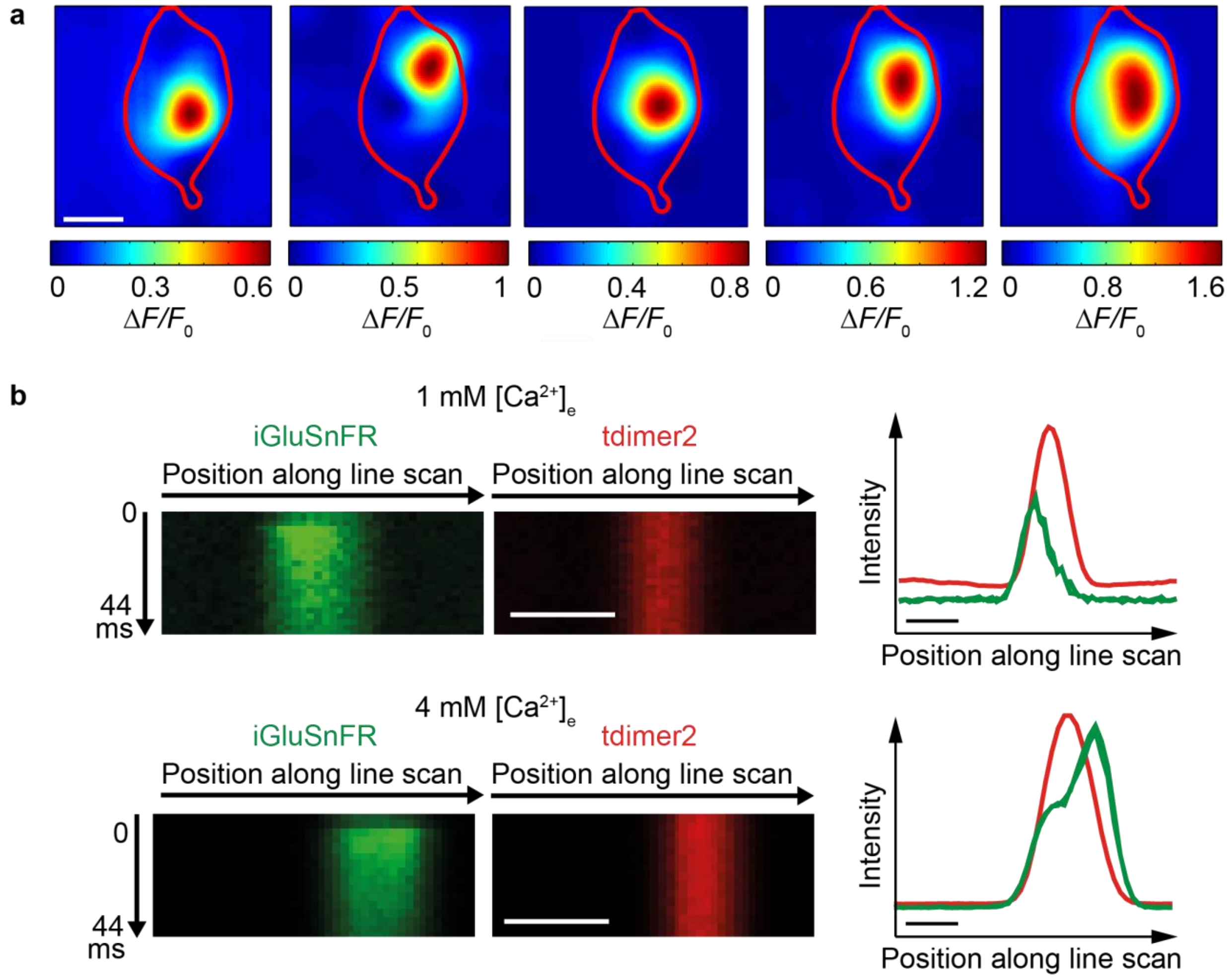

Supplementary Figure 4: Multisynaptic boutons show a second release site under high release probability conditions. a) Example of color-coded iGluSnFR signal of a multi-synapse bouton. Each frame represents the iGluSnFR signal in response to a single action potential. Scale bar, 1 µm. **b)** Example of the average intensity profile (average of ~ 30 trials) *post hoc* aligned on the red morphological marker of a straight-line scan across a multisynaptic bouton in 1 mM $[Ca^{2+}]_e$ (upper panel) with a single hot spot and in 4 mM $[Ca^{2+}]_e$ (lower panel) with the appearance of a second hot spot. Boutons with multiple release sites were rare; we excluded them from further analysis. Scale bar, 1 µm.

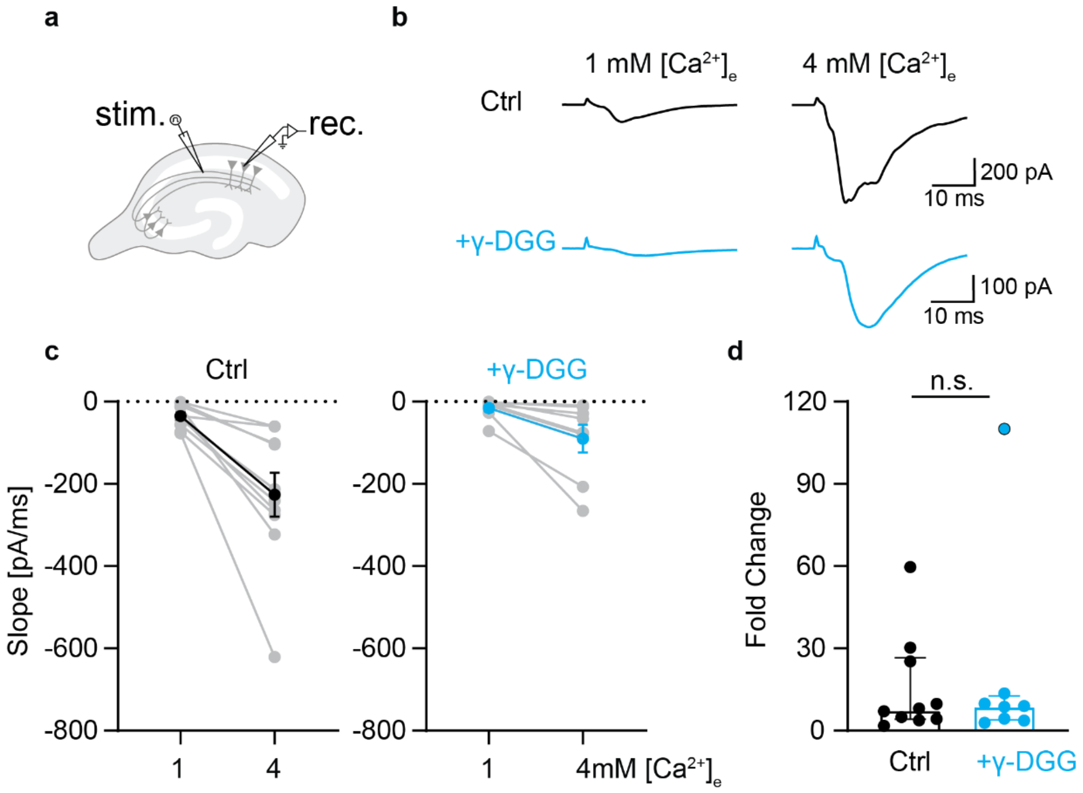

Supplementary Figure 5: Low occupancy of AMPARs. a) A monopolar electrode placed in *stratum radiatum* was used to elicit postsynaptic responses measured in whole cell voltage clamp from a CA1 neuron located ~300 µm away. To isolate AMPARs, the NMDAR blocker CPPene (10 µM) was added to the bath. **b)** Example traces of AMPARs responses measured in response to electrical field stimulation in control condition (upper traces) and in the presence of 10 mM γ-DGG, a competitive AMPARs antagonist (lower traces) which led to decreased evoked EPSCs. **c)** (Left panel) Slope measurements of evoked EPSCs in control condition in 1 mM $[Ca^{2+}]_e$: -35.1 ± 8.8 pA/ms and in 4 mM $[Ca^{2+}]_e$: -226.5 ± 53.2 pA/ms, *n* = 10 cells. (Right panel) Slope measurements upon application of γ-DGG in 1 mM $[Ca^{2+}]_e$: -15.5 ± 8.5 pA/ms and in 4 mM $[Ca^{2+}]_e$: -90.1 ± 33.7 pA/ms, *n* = 8 cells. Values are plotted as mean ± SEM. **d)** Decrease of AMPARs occupancy did not lead to a significant increase in postsynaptic fold change from low to high $[Ca^{2+}]_e$. There is no significant fold change from 1 mM to 4 mM $[Ca^{2+}]_e$ in the absence (median: 7.57-fold, IQR: 4.1–26.5-fold, *n* = 10 cells) or presence of γ-DGG (median: 8.84-fold, IQR: 3.8–12.6-fold, *n* = 8 cells) (two-sided Mann Whitney test, *p* = 0.97). Values are plotted as median with IQR. Source data are provided as a Source Data file.

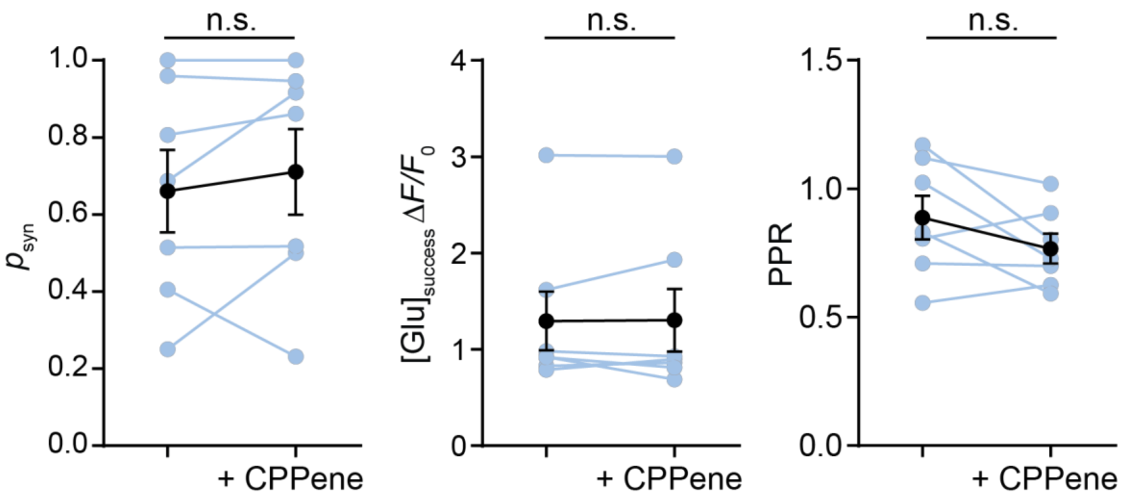

Supplementary Figure 6: Unchanged synaptic release probability upon block of NMDAR. iGluSnFR changes in fluorescence (single bouton, ~30 consecutive trials) were measured in response to single action potentials before and after the application of the NMDARs blocker CPPene (10 µM). There was no significant difference under NMDARs block in synaptic release probability, Ctrl: 0.66 ± 0.1, NMDAR block: 0.71 ± 0.1, paired t-test, $p = 0.41$; cleft glutamate, Ctrl: 1.3 ± 0.3 $\Delta F/F_0$, NMDAR block: 1.3 ± 0.3 $\Delta F/F_0$, two-sided Wilcoxon test, $p = 0.94$; and PPR, Ctrl: 89% ± 9%, NMDAR block: 77% ± 6%, paired t-test, $p = 0.13$; $n = 7$ boutons in 7 slices. Values are plotted as mean ± SEM. Source data are provided as a Source Data file.

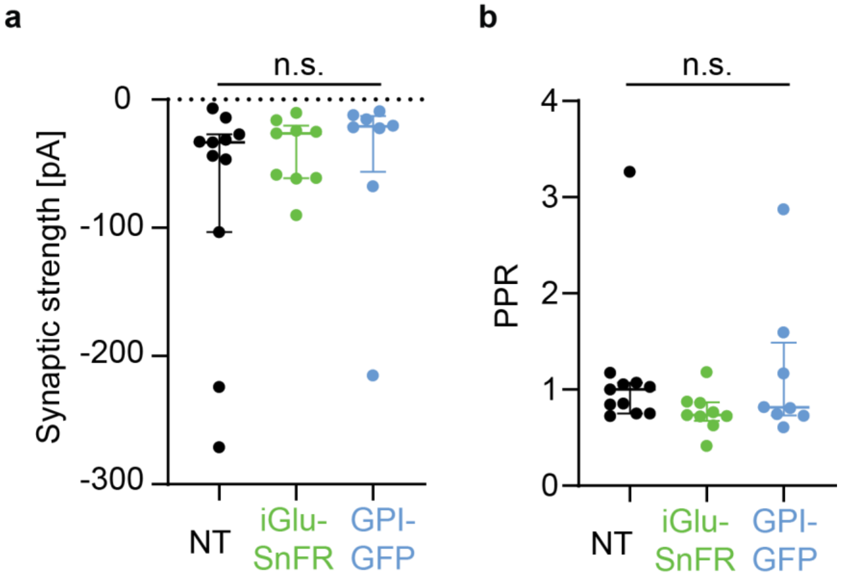

Supplementary Figure 7: Expression of iGluSnFR does not affect synaptic transmission. a) EPSCs were measured by dual patch-clamp recordings from connected CA3-CA1 pyramidal cell pairs in 4 mM $[Ca^{2+}]_e$. The NMDA blocker CPPene (10 µM) was added to the bath to isolate AMPAR responses. EPSCs were recorded from non-transfected (NT) CA3-CA1 pairs (median synaptic strength: -33.4 pA, IQR: -103.3 – -27.2 pA, n = 11 pairs); iGluSnFR-expressing CA3-CA1 pairs (median synaptic strength: -26.4 pA, IQR: -61.4 – -20.25 pA, n = 9 pairs); GPI-GFP-expressing CA3-CA1 pairs (median synaptic strength: -21.0 pA, IQR: -56.2 – -12.9 pA, n = 8 pairs). There was no significant difference in measured synaptic strength between the different groups of connected CA3-CA1 pairs (Kruskal-Wallis test, p = 0.31). **b)** EPSCs showed paired-pulse depression (ISI = 48 ms) in 4 mM $[Ca^{2+}]_e$ for NT CA3-CA1 pairs (median PPR: 100%, IQR: 75% – 107%, n = 11 pairs), for the iGluSnFR expressing CA3-CA1 (median PPR: 74%, IQR: 68% – 87%, n = 9 pairs) and for the GPI-GFP CA3 expressing-CA1 pairs (median PPR: 81%, IQR: 73%–149%, n = 8 pairs). There was no significant difference in measured synaptic strength between the different groups of connected CA3-CA1 pairs (Kruskal-Wallis test, p = 0.17). Values are plotted as median with IQR. Source data are provided as a Source Data file.

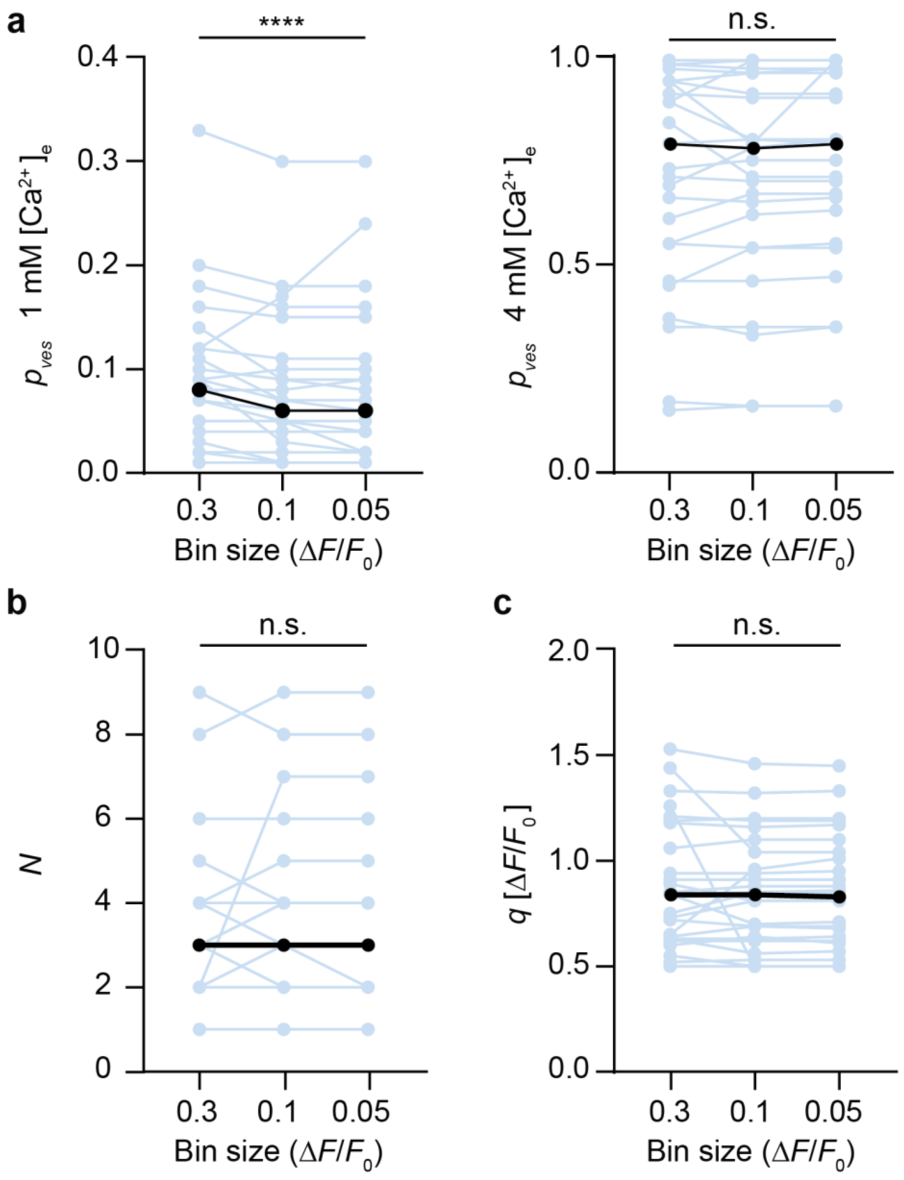

Supplementary Figure 8: Quantal analysis by fitting binomial distributions to histograms is robust with respect to histogram bin size.

a) p_{ves} in 1 mM $[Ca^{2+}]_e$ (left) and 4 mM $[Ca^{2+}]_e$ (right) extracted by binomial fitting of response amplitude histograms for different bin sizes. Median p_{ves} in 1 mM $[Ca^{2+}]_e$ was different for bin size 0.3 $\Delta F/F_0$, median: 0.08, IQR: 0.04-0.12, bin size 0.1 $\Delta F/F_0$, median: 0.06, IQR: 0.03 - 0.1 and bin size 0.05 $\Delta F/F_0$, median: 0.06, IQR: 0.02-0.1 (Friedman test, $p < 0.0001$ $n = 27$ boutons), but the size of the effect was small. p_{ves} in 4 mM $[Ca^{2+}]_e$ did not depend on bin size (one-way ANOVA, $p = 0.48$, $n = 27$ boutons). **b)** Number of vesicles N extracted by binomial fitting did not depend on bin size (Friedman test, $p = 0.97$, $n = 27$ boutons). **c)** Quantal size q extracted by binomial fitting did not depend on bin size (one-way ANOVA, $p = 0.33$, $n = 27$ boutons). Median values are plotted in black and individual datapoints in blue. Source data are provided as a Source Data file.

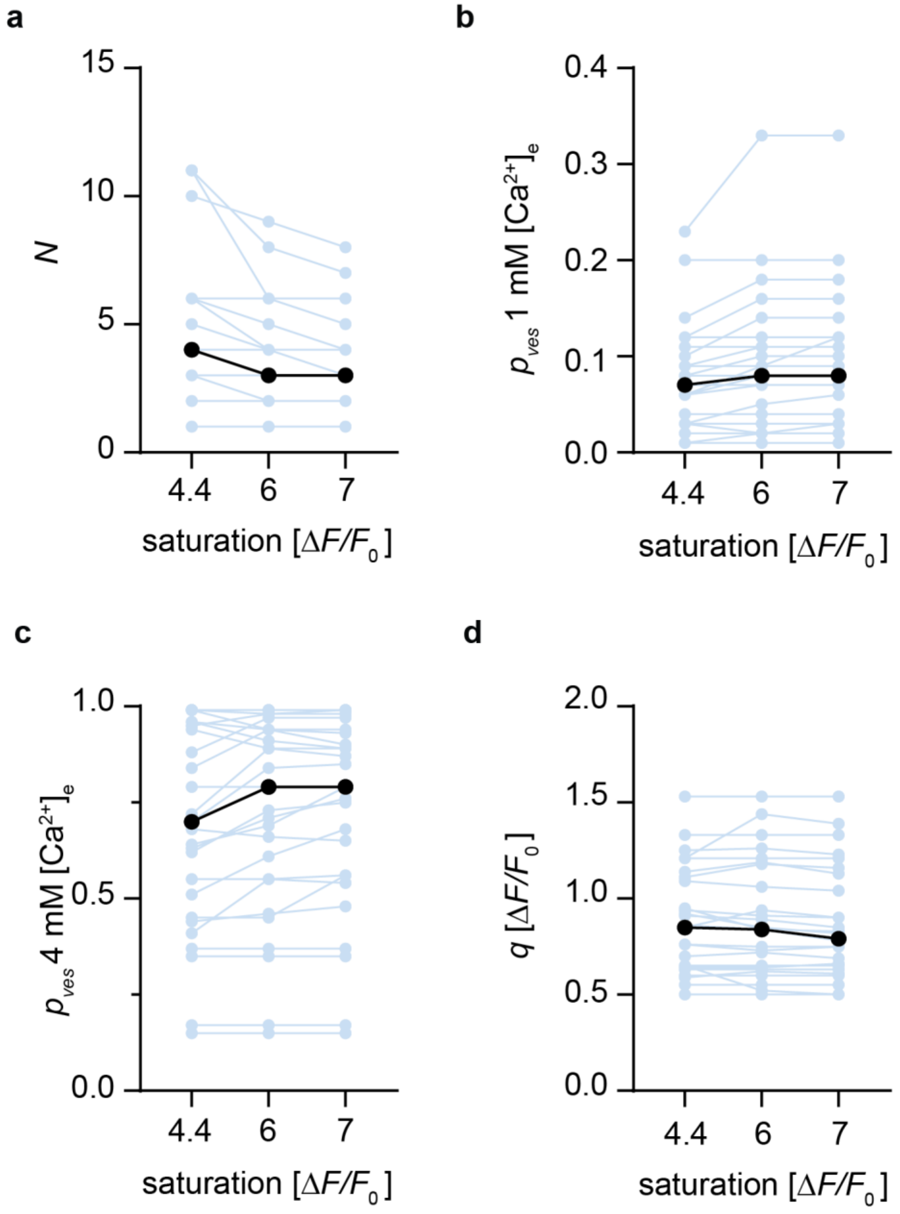

Supplementary Figure 9: Extracted parameters are robust to the assumed saturation value. Extracted number of **a)** vesicles **b)** vesicular release probability in 1 mM $[Ca^{2+}]_e$ **c)** vesicular release probability in 4 mM $[Ca^{2+}]_e$ and **d)** quantal size from the binomial fitting procedure of individual boutons assuming different saturation values of iGluSnFR-expressing boutons (n = 27 boutons). Median values are plotted in black. Our Monte-Carlo simulations (Fig. 5f) suggest apparent saturation at 6 $\Delta F/F_0$ for a synapse at optimal orientation and 7 $\Delta F/F_0$ for a 40° tilted synapse. Assuming saturation at 4.4 $\Delta F/F_0$, which is the literature value of iGluSnFR when calibrated with glutamate-containing solutions, requires one more vesicle (on average) to fit the shape of the response histograms. Source data are provided as a Source Data file.

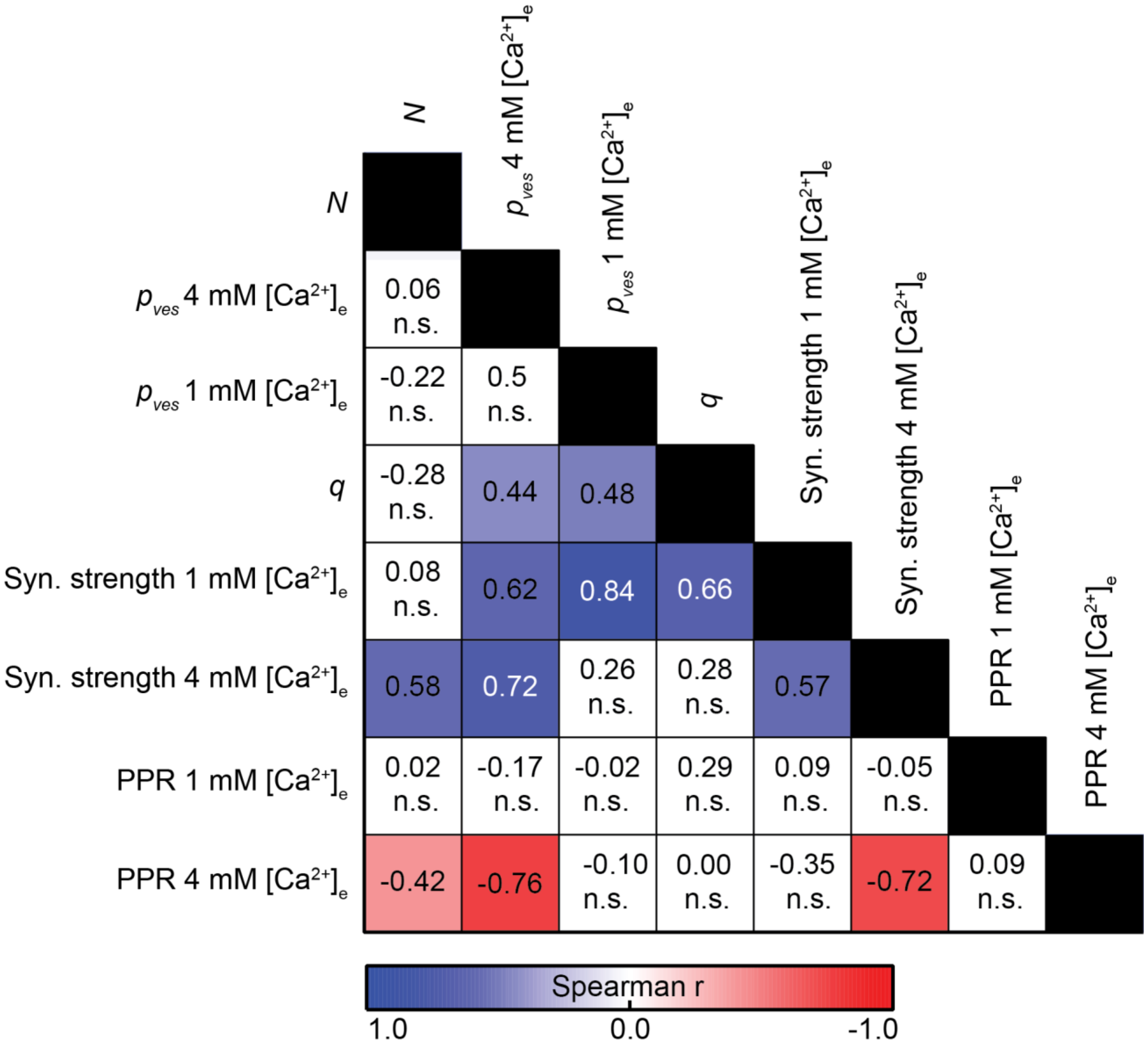

Supplementary Figure 10: Correlation matrix of extracted quantal parameters, synaptic strength (*p*q*N*) and paired-pulse ratio (PPR). Significant positive correlations are labeled in blue, significant negative correlations in red. Source data are provided as a Source Data file.

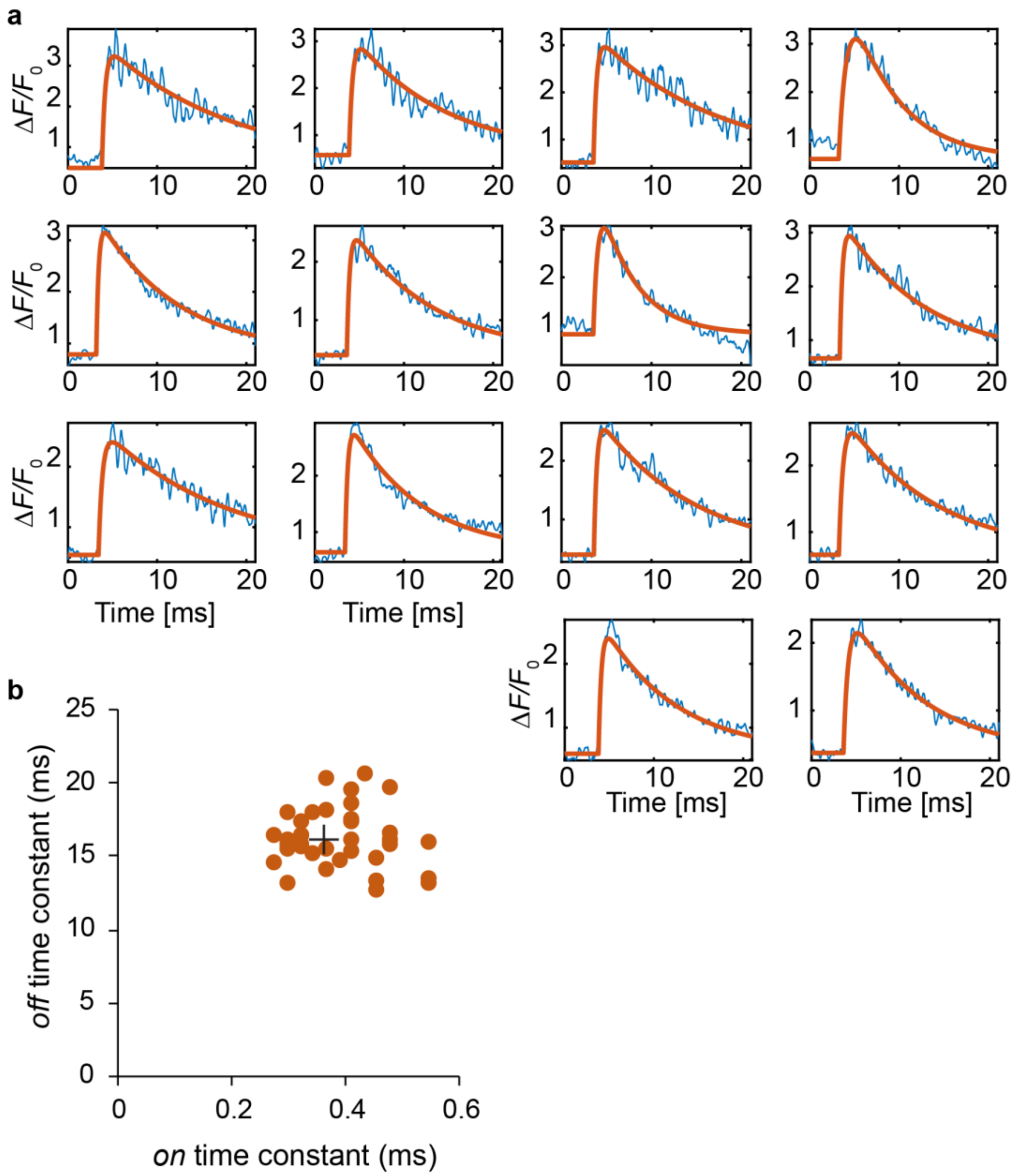

Supplementary Figure 11: Extracting iGluSnFR kinetics from synaptic imaging data.
a) Example traces of point scan (64 kHz sampling) of iGluSnFR signal in response to a single action potential. Blue lines: 14 success trials from 1 bouton. Red lines: double exponential fits.
b) Extracted time constants from n = 36 fits to traces from 3 boutons. Black cross: median τ_{on} (0.36 ms), median τ_{off} (16.1 ms). Reaction rates for the MCell model were based on these measurements (see Methods). Source data are provided as a Source Data file.
