# Supplementary material for: Vesicular release probability sets the strength of individual Schaffer collateral synapses
Source: Nat Commun. 2022 Oct 17;13:6126. doi: 10.1038/s41467-022-33565-6 (PMC9576736; doi:10.1038/s41467-022-33565-6)
Supplement: Supplementary file 3 — Reporting Summary [file 41467_2022_33565_MOESM3_ESM.pdf]

nature research

Corresponding author(s): Thomas Oertner

Last updated by author(s): Sep 9, 2022

Reporting Summary

Nature Research wishes to improve the reproducibility of the work that we publish. This form provides structure for consistency and transparency in reporting. For further information on Nature Research policies, see our Editorial Policies and the Editorial Policy Checklist.

Statistics

For all statistical analyses, confirm that the following items are present in the figure legend, table legend, main text, or Methods section.

n/a	Confirmed	
☐	☒	The exact sample size (n) for each experimental group/condition, given as a discrete number and unit of measurement
☐	☒	A statement on whether measurements were taken from distinct samples or whether the same sample was measured repeatedly
☐	☒	The statistical test(s) used AND whether they are one- or two-sided<br>*Only common tests should be described solely by name; describe more complex techniques in the Methods section.*
☒	☐	A description of all covariates tested
☐	☒	A description of any assumptions or corrections, such as tests of normality and adjustment for multiple comparisons
☐	☒	A full description of the statistical parameters including central tendency (e.g. means) or other basic estimates (e.g. regression coefficient) AND variation (e.g. standard deviation) or associated estimates of uncertainty (e.g. confidence intervals)
☐	☒	For null hypothesis testing, the test statistic (e.g. F, t, r) with confidence intervals, effect sizes, degrees of freedom and P value noted<br>*Give P values as exact values whenever suitable.*
☒	☐	For Bayesian analysis, information on the choice of priors and Markov chain Monte Carlo settings
☒	☐	For hierarchical and complex designs, identification of the appropriate level for tests and full reporting of outcomes
☒	☐	Estimates of effect sizes (e.g. Cohen's d, Pearson's r), indicating how they were calculated

Our web collection on statistics for biologists contains articles on many of the points above.

Software and code

Policy information about availability of computer code

Data collection	ScanImage 3.8 (Vidrio Technologies, LCC)<br>Ephus r2.1.0 (Vidrio Technologies, LCC)<br>MCell/CellBlender bundle 4.0.5 with Blender 2.93 (https://mcell.org/)
Data analysis	Custom-written Matlab code to extract quantal parameters is available on GitHub (https://github.com/toertner/optical-quantal-analysis) as well as the parameter files used for the MCell simulation (https://github.com/toertner/iGluSnFR-simulation). We used GraphPad Prism 8.4.3 and Excel (Version 1808) for statistical analysis.

For manuscripts utilizing custom algorithms or software that are central to the research but not yet described in published literature, software must be made available to editors and reviewers. We strongly encourage code deposition in a community repository (e.g. GitHub). See the Nature Research guidelines for submitting code & software for further information.

Data

Policy information about availability of data

All manuscripts must include a data availability statement. This statement should provide the following information, where applicable:

- Accession codes, unique identifiers, or web links for publicly available datasets
- A list of figures that have associated raw data
- A description of any restrictions on data availability

Parameter files used for the MCell simulation have been deposited on GitHub (https://github.com/toertner/iGluSnFR-simulation). The datasets generated and analyzed during the current study can be downloaded from the Zenodo repository (https://doi.org/10.5281/zenodo.6997652).

Field-specific reporting

Please select the one below that is the best fit for your research. If you are not sure, read the appropriate sections before making your selection.

☒ Life sciences ☐ Behavioural & social sciences ☐ Ecological, evolutionary & environmental sciences

For a reference copy of the document with all sections, see nature.com/documents/nr-reporting-summary-flat.pdf

Life sciences study design

All studies must disclose on these points even when the disclosure is negative.

Sample size	The number of optical measurements ('trials') from individual synapses was limited to 120-300 due to photodamage / indicator bleaching. Full optical quantal analysis was performed on 27 individual boutons. This study is the first attempt to extract quantal parameters from individual SC synapses in tissue. Since the variability of these parameters was unknown, we could not predetermine the sample size. We discovered highly significant correlations between specific quantal parameters, synaptic strength and paired-pulse ratio (Extended Data Fig. 10), indicating that our sample size was sufficient to draw statistically sound conclusions.
Data exclusions	Data exclusion criteria were 1) Loss of patch-clamp recording (somatic stimulation) before at least 60 + 60 trials were acquired; 2) Extremely low release probability in 1 mM Ca2+ (<2 successes out of 60 trials); 3) Excessive imaging noise (FWHM of imaging noise distribution > 0.4).
Replication	Quantal parameters were extracted from 27 individual boutons from 23 different animals (slice cultures). All replications allowed for successful extraction of parameters using our binomial fitting procedure. The extracted parameters were different in each experiment due to the variability of Schaffer collateral synapses.
Randomization	Boutons were chosen at random. High and low calcium conditions were applied in random sequence (cross-over design).
Blinding	As we did not compare different genotypes or treatments but characterized individual wild-type synapses, blinding was not applicable. The only manipulation that could have been performed blind are high vs low Ca2+ saline measurements. However, since the synaptic output is different by a factor of 10 under these conditions, the experimenter would have effectively unblinded herself after the first optical response.

Reporting for specific materials, systems and methods

We require information from authors about some types of materials, experimental systems and methods used in many studies. Here, indicate whether each material, system or method listed is relevant to your study. If you are not sure if a list item applies to your research, read the appropriate section before selecting a response.

Materials & experimental systems

n/a	Involved in the study	
☒	☐	Antibodies
☒	☐	Eukaryotic cell lines
☒	☐	Palaeontology and archaeology
☐	☒	Animals and other organisms
☒	☐	Human research participants
☒	☐	Clinical data
☒	☐	Dual use research of concern

Methods

n/a	Involved in the study	
☒	☐	ChIP-seq
☒	☐	Flow cytometry
☒	☐	MRI-based neuroimaging

Animals and other organisms

Policy information about studies involving animals; ARRIVE guidelines recommended for reporting animal research

Laboratory animals	male and female rat pups (Rattus norvegicus Wistar) were used to prepare organotypic cultures at postnatal day 4-6
Wild animals	the study did not involve wild animals
Field-collected samples	the study did not involve samples collected from the field
Ethics oversight	All procedures were performed in compliance with German law (Tierschutzgesetz der Bundesrepublik Deutschland, TierSchG) and according to the guidelines of Directive 2010/63/EU. Protocols were approved by the Behörde für Justiz und Verbraucherschutz (BJV) - Lebensmittelsicherheit und Veterinärwesen, Hamburg.

Note that full information on the approval of the study protocol must also be provided in the manuscript.
